# Enterovirus Sequence Data Obtained from Primate Samples in Central Africa Suggest a High Prevalence of Enteroviruses

**DOI:** 10.1128/MRA.00882-21

**Published:** 2021-12-09

**Authors:** Ipos Ngay Lukusa, Jean-Michel Takuo, Christelle Lumbu Banza, Joseph Le Doux Diffo, Placide Mbala Kingebeni, Nkom F. Ntumvi, Joseph Atibu Losoma, Ubald Tamoufe, Amethyst Gillis, Matthew LeBreton, James Ayukekbong, Damien O. Joly, Brad S. Schneider, Corina Monagin, Maria Makuwa, Nathan D. Wolfe, Edward M. Rubin, Jean-Jacques Muyembe-Tamfum, Christian E. Lange

**Affiliations:** a Metabiota Inc., Kinshasa, Democratic Republic of the Congo; b Metabiota Cameroon Ltd., Yaoundé, Centre Region, Cameroon; c Institut National de Recherche Biomédicale, Kinshasa, Democratic Republic of the Congo; d Metabiota Inc., San Francisco, California, USA; e Development Alternatives, Inc., Washington, DC, USA; f Mosaic, Yaoundé, Centre Region, Cameroon; g Metabiota Inc., Nanaimo, British Columbia, Canada; h Southbridge Care, Cambridge, Ontario, Canada; i Nyati Health Consulting, Nanaimo, British Columbia, Canada; j Etiologic, Oakland, California, USA; k Pinpoint Science, San Francisco, California, USA; l One Health Institute, School of Veterinary Medicine, University of California, Davis, Davis, California, USA; m Labyrinth Global Health, St. Petersburg, Florida, USA; KU Leuven

## Abstract

Enteroviruses infect humans and animals and can cause disease, and some may be transmitted across species barriers. We tested Central African wildlife and found *Enterovirus* RNA in primates (17) and rodents (2). Some sequences were very similar, while others were dissimilar to known species, highlighting the underexplored enterovirus diversity in wildlife.

## ANNOUNCEMENT

The genus *Enterovirus* (family *Picornaviridae*) contains many diverse viruses that infect humans and cause disease, including poliomyelitis (human poliovirus), hand, foot, and mouth disease (human enterovirus 71), and the common cold (human rhinoviruses) (1). Enteroviruses associated with many other mammalian species have also been discovered, but their diversity, distribution, and roles in disease are overall poorly understood (2, 3). As zoonotic transmission from animals in close contact with humans is of concern, we were interested in the diversity of enteroviruses in wildlife in Cameroon and the Democratic Republic of the Congo (DRC).

Samples from 1,450 bats, 488 rodents, 86 nonhuman primates (NHPs), and 65 shrews were collected in Cameroon and the DRC from 2003 to 2014. The samples included primarily oral and rectal swabs, liver and spleen tissue, as well as feces, and were obtained from animals that were trapped and released, animals in captivity, and animals hunted for consumption. RNA was isolated and reverse transcribed (4), before the samples were screened for enterovirus RNA using a family level consensus PCR targeting the 5′ noncoding region (5). Both strands of the PCR amplicons were sequenced (Sanger), aligned (ClustalW, Geneious 11.1.3), and subjected to phylogenetic analysis using MrBayes 3.2, employing default parameters and 4 chains of 1,000,000 generations, with final split frequencies below 0.01 (6). The first 10% of the trees was discarded, and the remaining trees were combined using TreeAnnotator (BEAST 2.5.1) and displayed using FigTree 1.4.4 (7, 8). Samples for which no RNA of the expected size could be amplified and sequenced were counted as negative.

**FIG 1:**
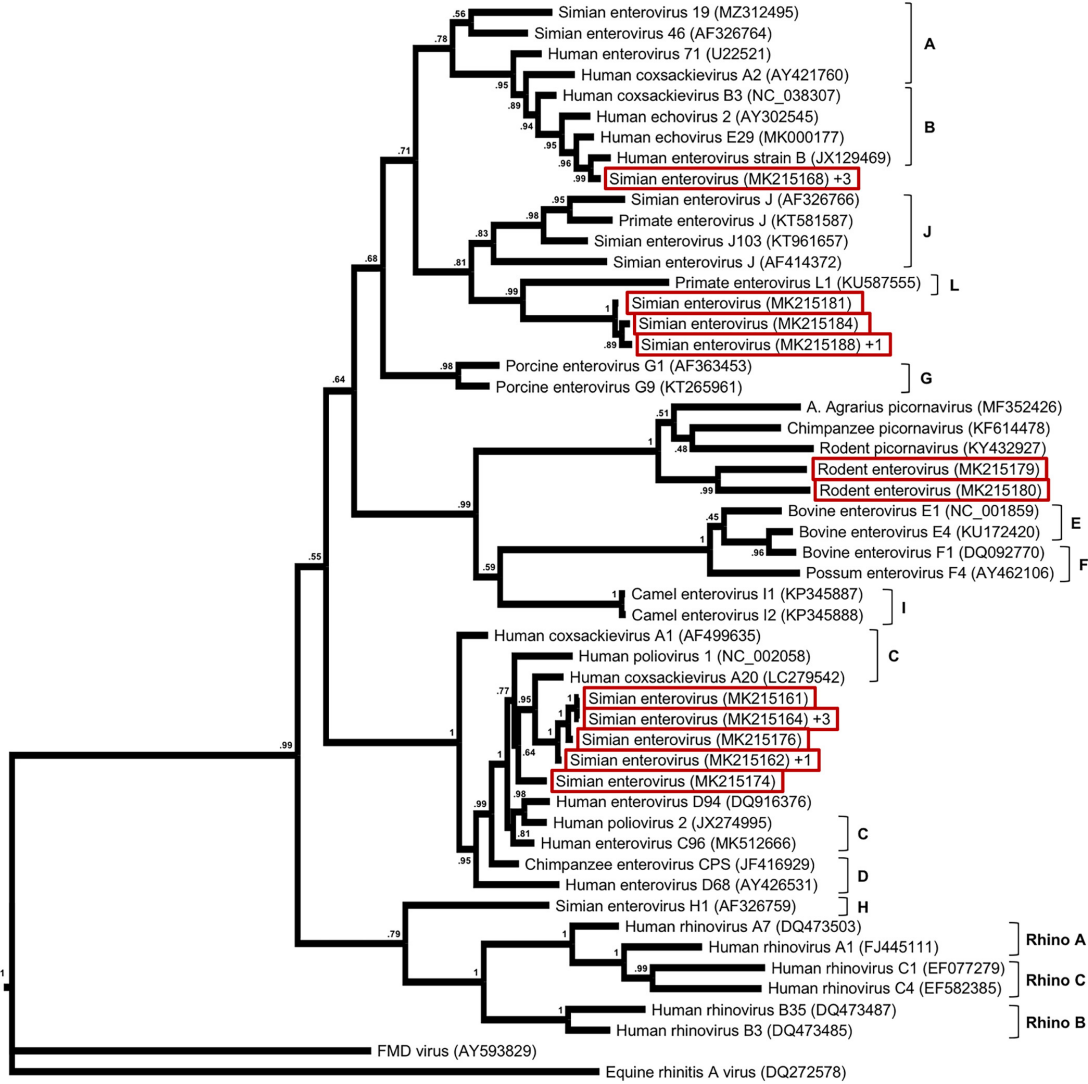
Maximum likelihood phylogenetic tree of *Enterovirus* sequences, based on the PCR-targeted 362-nucleotide sequence of the 5′ untranslated region (UTR). The tree includes the sequences detected during the project (red boxes) and the sequences of known species. The latter were selected to represent all classified species and include sequences with the highest similarities to the novel ones. As the tree is based on the partial 5′ UTR, its structure differs from trees based on the full genome or individual coding sequences. The numbers at the nodes indicate the bootstrap support. Novel sequences with high similarity (nucleotide identities of >97%) to other novel sequences are not included but are represented by a single sequence and “+*N*.” These are the sequences with GenBank accession numbers MK215173, MK215177, and MK215178 (represented by MK215168); MK215192 (represented by MK215188); MK215161, MK215165, and MK215167 (represented by MK215164); and MK215163 (represented by MK215162). The ICTV classification of species within the genus *Enterovirus* is indicated where applicable.

*Enterovirus* RNA was detected in 17 NHPs and 2 rodents (Table 1). The sequences fall into four phylogenetic clusters, one of them coinciding with the species enterovirus B, one clustering with enterovirus C and D sequences, one related to enterovirus L, and one clustering with unclassified enteroviruses from rodent and primate hosts (Fig. 1; Table 1).

**TABLE 1:** Sequencing and phylogenetic analysis data

Sample	GenBank accession no.	Amplicon size (nt)^*a*^	BLASTN search results^*b*^		Host (sample type)	Country (interface)
			Similarity (%)	Reference strain (GenBank accession no.)		
CD116032	MK215161	360	93	Coxsackievirus A13 (MG571836)	Pan troglodytes (feces)	Democratic Republic of the Congo (captive)
CD116033	MK215162	363	93	Coxsackievirus A17 (JF260925)	Pan troglodytes (feces)	Democratic Republic of the Congo (captive)
CD116035	MK215163	358	94	Coxsackievirus A17 (JF260925)	Pan troglodytes (feces)	Democratic Republic of the Congo (captive)
CD116037	MK215164	360	93	Coxsackievirus A13 (MG571836)	Pan troglodytes (feces)	Democratic Republic of the Congo (captive)
CD116038	MK215165	360	93	Coxsackievirus A13 (MG571836)	Pan troglodytes (feces)	Democratic Republic of the Congo (captive)
CD116040	MK215167	358	93	Coxsackievirus A13 (MG571836)	Pan troglodytes (feces)	Democratic Republic of the Congo (captive)
CD116055	MK215168	358	95	Human enterovirus strain B (JX129469)	Pan troglodytes (feces)	Democratic Republic of the Congo (captive)
CD116064	MK215173	358	96	Human enterovirus strain B (HM209138)	Pan troglodytes (feces)	Democratic Republic of the Congo (captive)
CD116066	MK215174	359	94	Coxsackievirus A24 (EF026081)	Pan troglodytes (feces)	Democratic Republic of the Congo (captive)
CD116072	MK215175	359	93	Coxsackievirus A13 (MG571836)	Pan troglodytes (feces)	Democratic Republic of the Congo (captive)
CD116079	MK215176	358	93	Coxsackievirus A13 (JF260920)	Pan troglodytes (feces)	Democratic Republic of the Congo (captive)
CD116084	MK215177	358	95	Human enterovirus strain B (JX129469)	Pan troglodytes (feces)	Democratic Republic of the Congo (captive)
CD116086	MK215178	358	95	Human enterovirus strain B (JX129469)	Pan troglodytes (feces)	Democratic Republic of the Congo (captive)
ECO05844	MK215179	321	76	*Picornaviridae* sp. (KF614478)	Praomys sp. (liver, spleen)	Cameroon (free-ranging)
ECO05846	MK215180	309	74	Apodemus agrarius picornavirus strain Longquan-Aa118 (MF352426)	*Praomys* sp. (liver, spleen)	Cameroon (free-ranging)
ECO50936	MK215181	358	83	Human enterovirus A (HM209159)	Cercopithecus nictitans (colon)	Cameroon (captive)
ECO50937	MK215184	358	83	Human enterovirus A (HM209159)	Allochrocebus preussi (small intestine)	Cameroon (captive)
ECO50938	MK215188	358	82	Uncultured enterovirus clone 0626416 (EU672963)	*Allochrocebus preussi* (colon)	Cameroon (captive)
ECO50939	MK215192	358	82	Uncultured enterovirus clone 0626416 (EU672963)	*Cercopithecus nictitans* (small intestine)	Cameroon (captive)

a nt, nucleotides.

b BLASTN search conducted on 26 October 2021.

The detection of *Enterovirus* RNA in almost 20% of the sampled NHPs supports previous findings that suggest a high prevalence of enteroviruses among primates (9–16). Even though attempts with multiple assays failed to produce sequence beyond the 5′ noncoding region, the results suggest that the diversity of NHP enteroviruses needs further exploration. Enteroviruses can be transmitted between humans and NHPs, and contact between these two is not uncommon across many parts of Central Africa, which is of concern (12, 13). The RNAs detected in the rodents suggests the presence of two novel enterovirus species, given their low sequence similarity and phylogenetic placement; however, in the absence of full genomic sequence information, classification is not possible. Despite having tested many bats in the study, we did not detect enterovirus RNA in any of them. Bats, which are hosts of many zoonotic viruses, including rabies and coronaviruses, can be experimentally infected with enteroviruses, but reports of genuine bat enteroviruses are sparse, unlike reports of other bat picornaviruses (4, 8, 17–22). We conclude that Central African bats may either not host many enteroviruses or that the enteroviruses that infect bats are genetically divergent enough from the known species to evade PCR detection with the primers used in this study.

### Data availability.

The partial genomic sequences described are deposited in GenBank under accession numbers MK215161 to MK215165, MK215167, MK215168, MK215173 to MK215181, MK215184, MK215188, and MK215192. The raw data from the collected samples and sampling maps are available at the Zenodo repository (https://zenodo.org/record/5528104).

## References

[1] Tapparel C, Siegrist F, Petty TJ, Kaiser L. 2013. Picornavirus and enterovirus diversity with associated human diseases. Infect Genet Evol 14:282–293. doi:10.1016/j.meegid.2012.10.016. 23201849

[2] Ayukekbong J, Kabayiza JC, Lindh M, Nkuo-Akenji T, Tah F, Bergström T, Norder H. 2013. Shift of Enterovirus species among children in Cameroon—identification of a new enterovirus, EV-A119. J Clin Virol 58:227–232. doi:10.1016/j.jcv.2013.07.005. 23895932

[3] Sadeuh-Mba SA, Bessaud M, Joffret ML, Endegue Zanga MC, Balanant J, Mpoudi Ngole E, Njouom R, Reynes JM, Delpeyroux F, Rousset D. 2014. Characterization of enteroviruses from non-human primates in Cameroon revealed virus types widespread in humans along with candidate new types and species. PLoS Negl Trop Dis 8:e3052. doi:10.1371/journal.pntd.0003052. 25079078 PMC4117447

[4] Kumakamba C, Niama FR, Muyembe F, Mombouli JV, Kingebeni PM, Nina RA, Lukusa IN, Bounga G, N'Kawa F, Nkoua CG, Atibu Losoma J, Mulembakani P, Makuwa M, Tamufe U, Gillis A, LeBreton M, Olson SH, Cameron K, Reed P, Ondzie A, Tremeau-Bravard A, Smith BR, Pante J, Schneider BS, McIver DJ, Ayukekbong JA, Hoff NA, Rimoin AW, Laudisoit A, Monagin C, Goldstein T, Joly DO, Saylors K, Wolfe ND, Rubin EM, Bagamboula MPassi R, Muyembe Tamfum JJ, Lange CE. 2021. Coronavirus surveillance in wildlife from two Congo basin countries detects RNA of multiple species circulating in bats and rodents. PLoS One 16:e0236971. doi:10.1371/journal.pone.0236971. 34106949 PMC8189465

[5] Wiyatno A, Antonjaya U, Ma'roef CN, Riswari SF, Djauhari H, Artika IM, Monagin C, Schneider BS, Myint KS, Alisjahbana B, Safari D, Kosasih H. 2016. Detection and identification of coxsackievirus B3 from sera of an Indonesian patient with undifferentiated febrile illness. J Infect Dev Ctries 10:880–883. doi:10.3855/jidc.7573. 27580335

[6] Ronquist F, Teslenko M, van der Mark P, Ayres DL, Darling A, Höhna S, Larget B, Liu L, Suchard MA, Huelsenbeck JP. 2012. MrBayes 3.2: efficient Bayesian phylogenetic inference and model choice across a large model space. Syst Biol 61:539–542. doi:10.1093/sysbio/sys029. 22357727 PMC3329765

[7] Bouckaert R, Vaughan TG, Barido-Sottani J, Duchêne S, Fourment M, Gavryushkina A, Heled J, Jones G, Kühnert D, De Maio N, Matschiner M, Mendes FK, Müller NF, Ogilvie HA, Du Plessis L, Popinga A, Rambaut A, Rasmussen D, Siveroni I, Suchard MA, Wu CH, Xie D, Zhang C, Stadler T, Drummond AJ. 2019. BEAST 2.5: an advanced software platform for Bayesian evolutionary analysis. PLoS Comput Biol 15:e1006650. doi:10.1371/journal.pcbi.1006650. 30958812 PMC6472827

[8] Cameron KN, Niama FR, Hayes B, Mbala P, Olson SH, Takuo JM, Ondzie A, Diffo JLD, Smith BR, Pante J, Laudisoit A, LeBreton M, Tamufe U, Makuwa M, Joly DO, Goldstein T, Muyembe Tamfum JJ, Bagamboula MPassi R, Lange CE. 2021. Sequences of previously unknown rhabdoviruses detected in bat samples from the Republic of the Congo. Vector Borne Zoonotic Dis 21:552–555. doi:10.1089/vbz.2020.2736. 34010076

[9] Hoffert WR, Bates ME, Cheever FS. 1958. Study of enteric viruses of simian origin. Am J Hyg 68:15–30. 13559208 10.1093/oxfordjournals.aje.a119946

[10] Fuentes-Marins R, Rodriguez AR, Kalter SS, Hellman A, Crandell RA. 1963. Isolation of enteroviruses from the “normal” baboon (Papio doguera). J Bacteriol 85:1045–1050. doi:10.1128/jb.85.5.1045-1050.1963. 14043993 PMC278282

[11] Nix WA, Jiang B, Maher K, Strobert E, Oberste MS. 2008. Identification of enteroviruses in naturally infected captive primates. J Clin Microbiol 46:2874–2878. doi:10.1128/JCM.00074-08. 18596147 PMC2546737

[12] Harvala H, Sharp CP, Ngole EM, Delaporte E, Peeters M, Simmonds P. 2011. Detection and genetic characterization of enteroviruses circulating among wild populations of chimpanzees in Cameroon: relationship with human and simian enteroviruses. J Virol 85:4480–4486. doi:10.1128/JVI.02285-10. 21345956 PMC3126250

[13] Harvala H, Van Nguyen D, McIntyre C, Ahuka-Mundeke S, Ngole EM, Delaporte E, Peeters M, Simmonds P. 2014. Co-circulation of enteroviruses between apes and humans. J Gen Virol 95:403–407. doi:10.1099/vir.0.059048-0. 24189620 PMC4093782

[14] Van Nguyen D, Harvala H, Ngole EM, Delaporte E, Woolhouse ME, Peeters M, Simmonds P. 2014. High rates of infection with novel enterovirus variants in wild populations of mandrills and other Old World monkey species. J Virol 88:5967–5976. doi:10.1128/JVI.00088-14. 24623420 PMC4093852

[15] Mombo IM, Berthet N, Lukashev AN, Bleicker T, Brünink S, Léger L, Atencia R, Cox D, Bouchier C, Durand P, Arnathau C, Brazier L, Fair JN, Schneider BS, Drexler JF, Prugnolle F, Drosten C, Renaud F, Leroy EM, Rougeron V. 2015. First detection of an enterovirus C99 in a captive chimpanzee with acute flaccid paralysis, from the Tchimpounga Chimpanzee Rehabilitation Center, Republic of Congo. PLoS One 10:e0136700. doi:10.1371/journal.pone.0136700. 26301510 PMC4547728

[16] Mombo IM, Lukashev AN, Bleicker T, Brünink S, Berthet N, Maganga GD, Durand P, Arnathau C, Boundenga L, Ngoubangoye B, Boué V, Liégeois F, Ollomo B, Prugnolle F, Drexler JF, Drosten C, Renaud F, Rougeron V, Leroy E. 2017. African non-human primates host diverse enteroviruses. PLoS One 12:e0169067. doi:10.1371/journal.pone.0169067. 28081564 PMC5233426

[17] Reagan RL, Delaha EC, Cook SR, Brueckner AL. 1954. Response of the cave bat (Myotus lucifugus) to the Lansing strain of poliomyelitis virus. Cornell Vet 44:449–452. 13200093

[18] Reagan RL, Schenck DM, Brueckner AL. 1952. Viability of the Lansing strain of poliomyelitis virus in the bat (Eptesicus fuscus). Proc Soc Exp Biol Med 80:257–259. doi:10.3181/00379727-80-19587. 14949015

[19] Dempster G, Grodums EI, Spencer WA. 1961. Experimental Coxsackie B-3 infection in the hibernating squirrel and bat. Can J Microbiol 7:587–594. doi:10.1139/m61-068. 13721808

[20] Lukashev AN, Corman VM, Schacht D, Gloza-Rausch F, Seebens-Hoyer A, Gmyl AP, Drosten C, Drexler JF. 2017. Close genetic relatedness of picornaviruses from European and Asian bats. J Gen Virol 98:955–961. doi:10.1099/jgv.0.000760. 28555547

[21] Yinda CK, Zell R, Deboutte W, Zeller M, Conceição-Neto N, Heylen E, Maes P, Knowles NJ, Ghogomu SM, Van Ranst M, Matthijnssens J. 2017. Highly diverse population of Picornaviridae and other members of the Picornavirales, in Cameroonian fruit bats. BMC Genomics 18:249. doi:10.1186/s12864-017-3632-7. 28335731 PMC5364608

[22] Anthony SJ, Johnson CK, Greig DJ, Kramer S, Che X, Wells H, Hicks AL, Joly DO, Wolfe ND, Daszak P, Karesh W, Lipkin WI, Morse SS, Mazet JAK, Goldstein T, PREDICT Consortium. 2017. Global patterns in coronavirus diversity. Virus Evol 3:vex012. doi:10.1093/ve/vex012. 28630747 PMC5467638

